# New Halogen-Containing Drugs Approved by FDA in 2021: An Overview on Their Syntheses and Pharmaceutical Use

**DOI:** 10.3390/molecules27051643

**Published:** 2022-03-02

**Authors:** Davide Benedetto Tiz, Luana Bagnoli, Ornelio Rosati, Francesca Marini, Luca Sancineto, Claudio Santi

**Affiliations:** Group of Catalysis, Synthesis and Organic Green Chemistry, Department of Pharmaceutical Sciences, University of Perugia, Via del Liceo 1, 06100 Perugia, Italy; luana.bagnoli@unipg.it (L.B.); ornelio.rosati@unipg.it (O.R.); francesca.marini@unipg.it (F.M.); luca.sancineto@unipg.it (L.S.)

**Keywords:** fluorine, chlorine, halogens, FDA, drugs

## Abstract

This review describes the recent Food and Drug Administration (FDA)-approved drugs (in the year 2021) containing at least one halogen atom (covalently bound). The structures proposed throughout this work are grouped according to their therapeutical use. Their synthesis is presented as well. The number of halogenated molecules that are reaching the market is regularly preserved, and 14 of the 50 molecules approved by the FDA in the last year contain halogens. This underlines the emergent role of halogens and, in particular, of fluorine and chlorine in the preparation of drugs for the treatment of several diseases such as viral infections, several types of cancer, cardiovascular disease, multiple sclerosis, migraine and inflammatory diseases such as vasculitis.

## 1. Introduction

In the last years, new halogen-containing drugs have emerged. In 2021, 14 new chemical entities (Table S1) were approved by the FDA for clinical use [1]. In the previous year, the very same number of halogenated molecules reached the market [2]. These data highlight two aspects: from one side, a big effort in searching for new therapies has been done despite the COVID-19 pandemic, and from the other, the use of halogens is becoming regular in medicinal chemistry.

**Figure 1:**
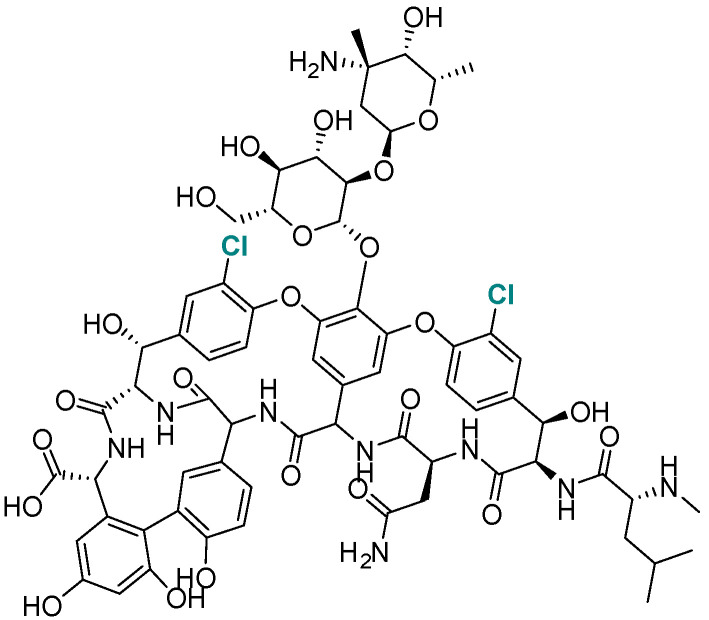
Structure of vancomycin, a natural antibacterial compound with activity against methicillin-resistant *Staphylococcus aureus* (MRSA).

Not only synthetic compounds but also halogenated natural products are worth mentioning, since they display a broad range of biological activities (e.g., antibacterial, antifungal and anticancer) [3]. As an example, vancomycin (Figure 1) is a clinical, chlorine-containing antibiotic obtained from the bacterium *Streptomyces orientalis*, which is mainly used to treat methicillin-resistant *Staphylococcus aureus* (MRSA) infections [4]. 

Another very important class is marine algae that have been largely investigated in the last few decades given their ability to produce halogenated metabolites with potential use, among the others, in the pharmaceutical industry [5]. In this particular case, chlorine and bromine appear often in biologically active metabolites, whereas iodine and fluorine remain quite unusual within the chemical structures [6]. It is interesting to note that bromine is more frequently present as a substituent in algae organohalogenated compounds in spite of chlorine being more concentrated than bromine in sea water [5]. 

Opposite to this trend, fluorine is prevalently employed in modern medicinal chemistry [7]: eight approved drugs out of 14 in 2021 were fluorine-containing drugs, four contained chlorine and two contained a combination of the two halogens. Thirteen new fluorinated drugs were approved by the FDA in 2020 for commercial use [8]. None possessed bromine or iodine. For this reason, we will confine our topic to fluorine and chlorine only. Only a few examples in the literature have reported fluorine in natural organisms. Fluoroacetate and some fluorinated fatty acids have been reported, for instance, in actinomycetes species [9].

In this review, we will briefly describe the chemical properties of halogen atoms in order to justify the predominance of -F and -Cl in drugs. Moreover, we will provide the chemical synthesis of the 14 approved halogenated drugs in 2021. 

## 2. Fluorine and Chlorine in Medicinal Chemistry

Despite the smallest size among the halogens, the introduction of fluorine onto a chemical scaffold is able to introduce changes that affect the physicochemical properties and the conformation of a molecule. Being the most electronegative element in the periodic table (xP (Pauling) 4.0) [10], fluorine plays an important role in the modulation of p*K*_a_ of neighboring functionalities [11]. For instance, the fluorination in a 3-piperidinylindole antipsychotic drugs series decreased the basicity of the neighboring amine, thereby improving the bioavailability and affinity towards the 5-HT_2A_ serotonin receptor [12]. This came from the fact that it was not necessary to further substitute piperidine in order to obtain good binding at the 5-HT_2A_ receptors. Further fluorination at position 6 of the indole provided higher metabolic stability [10,12] (Table 1). 

Lipophilicity is affected as well by the addition of fluorine onto aliphatic and aromatic scaffolds. In the former case, the monofluorination or trifluoromethylation of saturated alkyl groups usually decreases the lipophilicity due to the strong electron-withdrawing capabilities of fluorine [13]. In the latter, fluoro-arenes are more lipophilic than des-fluoro ones due to the low polarizability of the C-F bond [13]. 

Moreover, the installation of a fluorine atom in the ortho-position to an NH function on the aromatic ring is often used to enhance the membrane permeability [14].

From a conformational point of view, the addition of one single fluorine has a reduced steric effect, leaving mostly unchanged the interaction with the receptor site if compared to the same interaction with a molecule bearing a hydrogen atom in the same position; this can be explained by the similar van der Waals radii (r_v_) of fluorine and hydrogen: 1.47 Å and 1.20 Å, respectively. On the other hand, the commonly used trifluoromethyl group is sterically more demanding and almost equivalent to an ethyl group [15]. 

The highly electronegative nature of fluorine makes it a good hydrogen bond acceptor from H-bond donors but does not establish as good of halogen bonds as chlorine and bromine, because it does not typically feature a σ-hole [16].

Another important use is that fluorinated functionality can be incorporated into endogenous substrates or ligands through ^19^F-markers to investigate protein functions [17].

**Figure 2:**
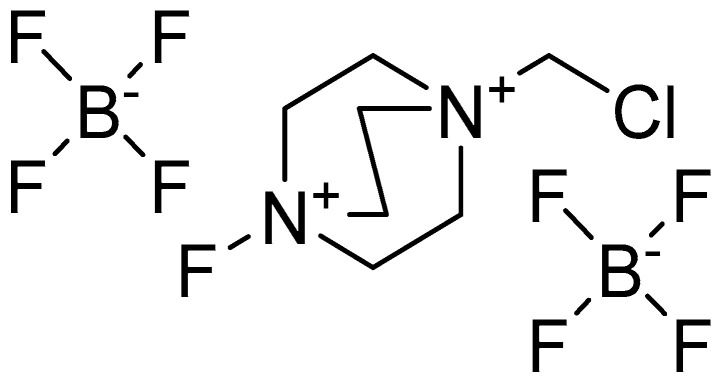
Structure of Selectfluor^®^.

Synthetically, given the high electronegativity of fluorine, the design of electrophilic fluorine source (F^+^) has been more challenging over the years with respect to bromine and chlorine sources. In this sense, Selectfluor^®^ [18] (Figure 2) has been groundbreaking, allowing several transformations on alkanes [19] and aromatic scaffolds [20]. In regard to the aromatic substrates, electron-donating substituents are known to increase the rate of the formation and yield of fluorinated compounds, obviously [21].

In summary, although it has a weak ability to form halogen bonds, fluorine establishes strong and stable bonds with carbon and allows fine-tuning several properties (both physicochemical and volumetric).

In 2019, more than 250 FDA-approved chlorine-containing drugs were available on the market [22]; this underlines the importance of this element in drug discovery.

According to Business Wire, chlorine is a major key ingredient in many drugs to treat diseases such as meningitis, cholera, plague, typhoid, bacterial skin infections, respiratory and nervous system problems, etc. [22].

Chlorine occupies an intermediate position in the halogen series. It is greater in size than fluorine, and its bond with carbon (C–Cl bond) is stable enough that it allows its insertion in diverse heterocyclics of pharmacological value [23]. Chlorine is a better halogen bond acceptor compared to fluorine. The most important impact of a nonreactive chlorine atom on the biological activity of many compounds comes when it is a substituent on an aromatic, heteroaromatic or olefinic moiety. In these cases, the steric and/or electronic effects of the chlorine substituents lead to local electronic attraction or repulsion and/or to steric interference with any surrounding amino acids of target proteins [22].

The chlorine atom is often viewed as isosteric and has similar physicochemical properties of the methyl group; therefore, it is very often selected as a bioisosteric replacement because of its ability to alter the in vivo metabolism. 

Another important aspect that needs to be considered is that the chemical processes for the synthesis of chlorine-containing compounds are well-known on the industrial scale and has reasonable costs.

## 3. New Halogen-Containing Drugs

In this section, we will discuss some synthetic aspects for the newly halogenated approved drugs in 2021 by grouping them following their therapeutic indication: cancer, viral infections, multiple sclerosis, migraines, vasculitis and cardiovascular diseases. 

### 3.1. Halogenated Anticancer Drugs Approved in 2021

In 2021, on the occasion of the 50th anniversary of the National Cancer Act [24], several new therapies were approved by the FDA (six halogenated compounds: tivozanib, sotorasib, melphalan flufenamide, asciminib, infrigatinib and umbralisib). We included in this group two more drugs that are not, strictly speaking, anticancer agents, but they are connected to the disease (Piflufolastat F-18 for the identification of prostate antigen-positive lesions in patients suspected of prostate cancer metastasis and belzutifan, which is used for von Hippel-Lindau disease, an inherited disorder whose hallmarks are tumors and cysts) [1]. 

#### 3.1.1. Tivozanib 

Tivozanib, (**10**, brand name Fotivda^®^) is intended for treating adults with advanced renal cell carcinoma. It works by blocking the activity of proteins known as VEGFR (Vascular Endothelial Growth Factor Receptor). Therefore, tivozanib limits cancer vascularization and, ultimately, the growth of the cancer [25]. 

**Scheme 1:**
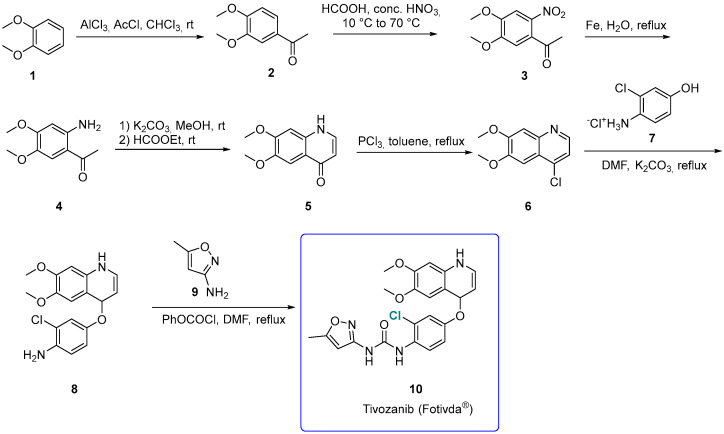
Synthesis of tivozanib (**10**) [27].

Compared to the earlier generation tyrosine kinase inhibitors, tivozanib was designed to optimize Vascular Endothelial Growth Factor Receptor (VEGFR) blockade while reducing off-target toxic effects, ultimately resulting in dose reductions [26]. The synthesis [27] (Scheme 1) starts from 1,2-dimethoxybenzene (**1**) that undergoes Friedel-Crafts acylation in chloroform. The so-formed ketone **2** was nitrated (**3**) and reduced the nitro functionality by using elemental iron at reflux (**4**). The subsequent ring closure by using ethyl formate afforded the substituted quinoline **5**, which was converted into the corresponding chloride (**6**) under PCl_3_ conditions. The nucleophilic substitution with 2-chloro-4-hydroxybenzenaminium chloride (**7**) afforded **8**, which was finally converted into tivozanib (**10**) by using phenyl chloroformate and 5-methylisoxazol-3-amine (**9**).

The reported yield for this pathway was 28.1%. A better, improved synthesis of tivozanib has been released. The major advantage is represented by the avoidance of nitric acid and of metallic iron (Scheme S1) [28]. 

#### 3.1.2. Sotorasib 

Sotorasib (**21**, brand name Lumakras^®^) is a Rat Sarcoma (RAS) GTPase family inhibitor developed by Amgen for the treatment of solid tumors with Kirsten Rat Sarcoma Viral Oncogene Homolog (KRAS) mutations, including non-small cell lung cancer (NSCLC) and colorectal cancer.

In a recent publication, the authors stated that the introduction of two aromatic fluorine led to an increase in Madin−Darby canine kidney (MDCK) cell trans well permeability assay [29].

**Scheme 2:**
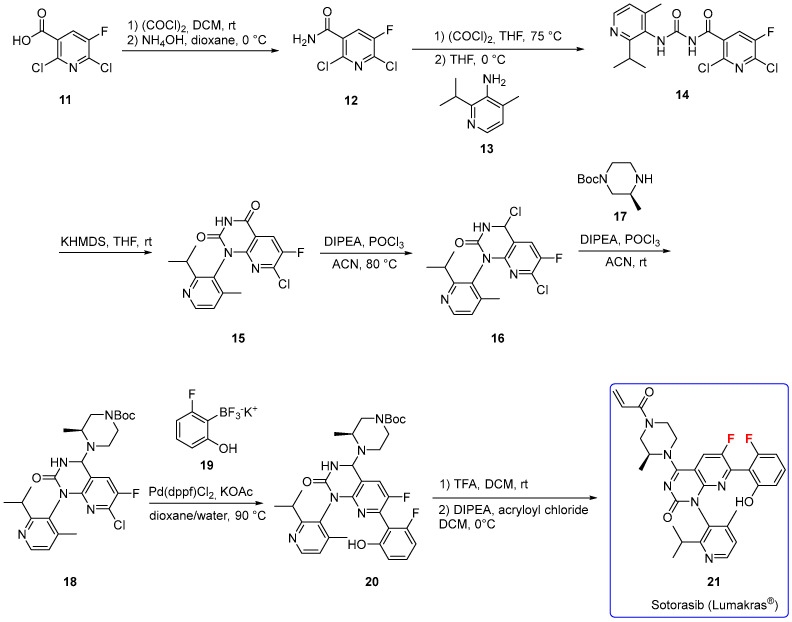
Synthesis of sotorasib (**21**) [30].

The synthesis [30] is shown in Scheme 2. 2,6-Dichloro-5-fluoronicotinic acid (**11**) was transformed into the corresponding primary amide **12** that was, in turn, converted into the urea **14** via using oxalyl chloride and the substituted aniline **13**. Compound **14** was cyclized upon the addition of Potassium (K) HexaMethylDiSilazide (KHMDS). Subsequently, the chlorination of **15** afforded **16**, which underwent nucleophilic substitution of the BOC-protected piperazine **17** to yield **18**. The Suzuki–Miyaura coupling between **18** and (2-fluoro-6-hydroxyphenyl) potassium trifluoroborate **19** promoted by Pd(dppf)Cl_2_ afforded the derivative **20**. Finally, BOC deprotection and the reaction with acryloyl chloride afforded sotorasib (**21**). An improved (a more scalable and efficient in yield) synthesis of sotorasib employing boroxine (**S9**, Figure S1) in the Suzuki–Miyaura step has been also reported [31]. 

#### 3.1.3. Melphalan Flufenamide 

Melphalan flufenamide (melflufen, **34**, brand name Pepaxto^®^) is a peptide-conjugated alkylating drug developed by Oncopeptides for the treatment of multiple myeloma (MM) and amyloid light-chain amyloidosis. 

Chemically, it is the ethyl ester of a lipophilic dipeptide consisting of melphalan and *para*-fluoro-l-phenylalanine [32]. 

**Scheme 3:**
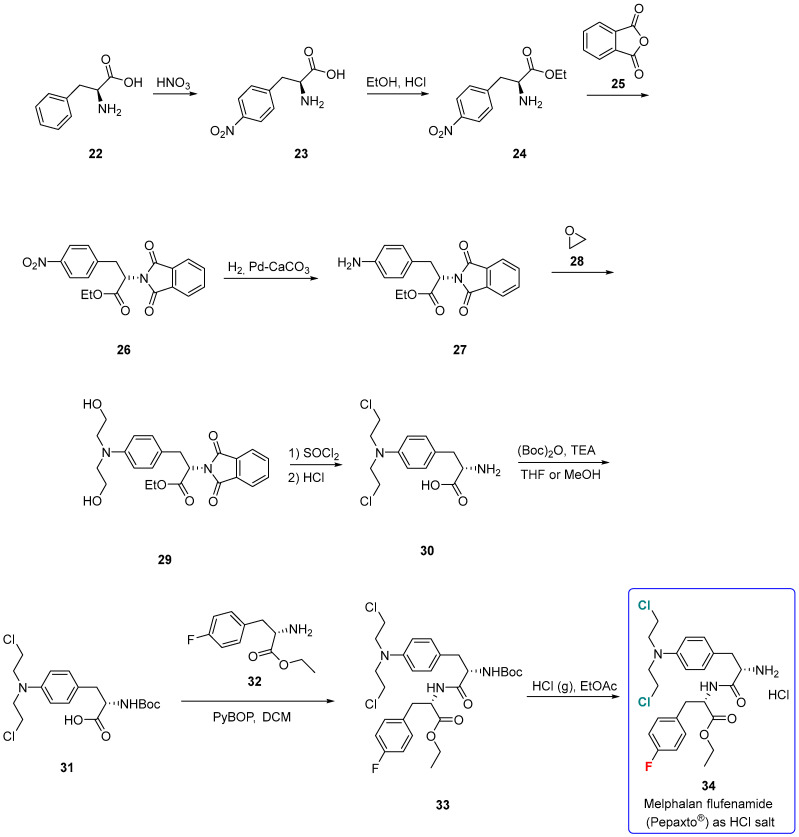
Synthesis of melphalan flufenamide hydrochloric salt (**34**) [33].

The chlorine atoms make the directly attached carbons electrophilic, thus rendering them a suitable site for endogenous nucleophiles (e.g., nitrogen from a guanine base), whereas the aromatic fluorine is important for pharmacokinetic properties. The synthesis [33] is shown in Scheme 3. The initial step is the nitration of l-phenylalanine (**22**), which gives 4-nitro-l-phenylalanine (**23**). The Fischer esterification on **23** afforded the ethyl ester **24**, whose amino group was protected with phthalic anhydride (**25**) to yield **26**. The reduction of the nitro group, using palladium on calcium carbonate as a catalyst, afforded the aniline derivative **27**. The subsequent alkylation with ethylene oxide (**28**) formed a bis-(2-hydroxyethyl)-amino derivative (**29**) whose alcoholic functions were converted to chlorine atoms by using thionyl chloride. The removal of phthalimide protection was carried out using HCl, affording the chlorinated derivative **30**. The amino group of **30** was protected with BOC to generate amino acid derivative **31**, which was condensed with *para*-fluoro-l-phenylalanine (**32**) using the peptide coupling reagent PyBOP to afford **33**.

Lastly, the removal of the BOC-protecting group by adding gaseous HCl yielded melflufen hydrochloride (**34**). A multi-kilogram production route (Scheme S2) for melflufen in high purity has been recently disclosed [34].

#### 3.1.4. Asciminib

Asciminib (**42**, brand name Scemblix^®^) was approved in 2021 for patients with Philadelphia chromosome-positive chronic myeloid leukemia [35]. Asciminib is an allosteric protein kinase inhibitor [36]. 

From X-ray studies, it is visible that one fluorine atom makes a highly directional polar interaction with the carbonyl carbon of leucine L359 deep in the pocket of the target Breakpoint Cluster Region-c-abl Oncogene 1 (BCR-ABL1) oncoprotein [36].

**Scheme 4:**
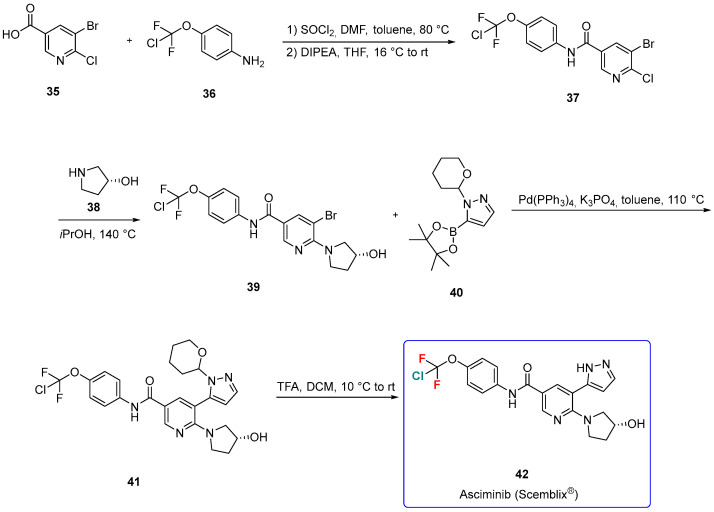
Synthesis of asciminib (**42**) [37].

The synthesis [37] of asciminib (Scheme 4) starts from 5-bromo-6-chloronicotinic acid (**35**), which is functionalized to the amide **37** via activation of the carboxylic acid by SOCl_2_ and subsequent nucleophilic substitution with 4-(chlorodifluoromethoxy)aniline (**36**). Compound **37** is then treated with (*R*)-pyrrolidin-3-ol (**38**) in a S_N_Ar reaction to afford **39**. A Suzuki–Miyaura coupling between **39** and the boronic ester (tetrahydropyran-protected at the pyrazolic nitrogen) **40** catalyzed by Palladium-tetrakis(triphenylphosphine) afforded **41**. Finally, the deprotection of tetrahydropyran protecting group gave asciminib (**42**).

**Scheme 5:**
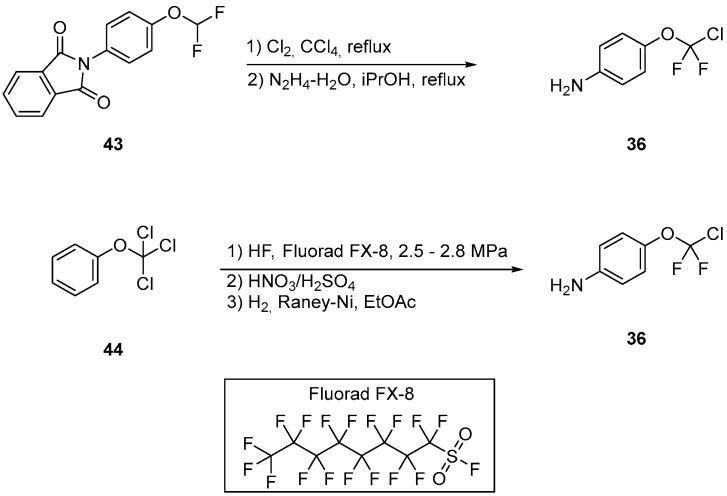
Possible methods for the preparation of synthon **36 [39]**.

Regarding the preparation of halogenated synthon **36** (Scheme 5), two main routes have been disclosed. The first employs the phthalimido-protected 4-(difluoromethoxy)aniline (**43**), followed by chlorination by molecular chlorine and removal of the phthalimide protecting group by hydrazine hydrate [38]. The second involves direct fluorination (by HF and catalyst Fluorad FX 8) at high pressure on (trichloromethoxy)benzene (**44**), followed by nitration with HNO_3_ and the reduction of the nitro group with H_2_/Raney-Ni [39]. 

#### 3.1.5. Infigratinib

Infigratinib (**50**, brand name Truseltiq^®^) is a fibroblast growth factor receptor (FGFR)-specific tyrosine kinase inhibitor being codeveloped by QED Therapeutics and Helsinn for the treatment of cholangiocarcinoma, urothelial carcinoma and other FGFR-driven conditions [40]. 

**Scheme 6:**
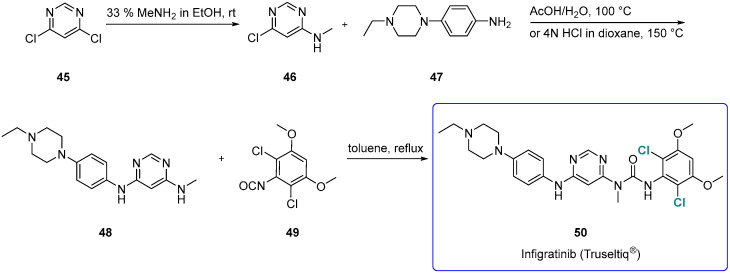
Synthesis of infigratinib (**50**) [42].

A Structure Activity Relationship (SAR) study established a preference for 2,6-dichloro or 3,5-dimethoxy substitutions at the NH-phenyl region to impart selectivity and potency [41]. The synthesis [42] (Scheme 6) starts from 4,6-dichloropyrimidine (**45**), which is treated with ethanolic methylamine (33%) to afford the mono-chloro-substituted pyrimidine **46** that is, in turn, converted to diaminopyrimidine **48** by adding the appropriate 4-(4-ethylpiperazin-1-yl)aniline (**47**) in acetic acid or HCl/dioxane as a solvent. The HCl/dioxane system was demonstrated to be a convenient alternative to acetic acid when the acetylation of the aniline is predominant in the desired substitution of the chlorine atom [42].

Lastly, **48** was reacted with the isocyanate **49** to afford Infigratinib (**50**).

**Scheme 7:**
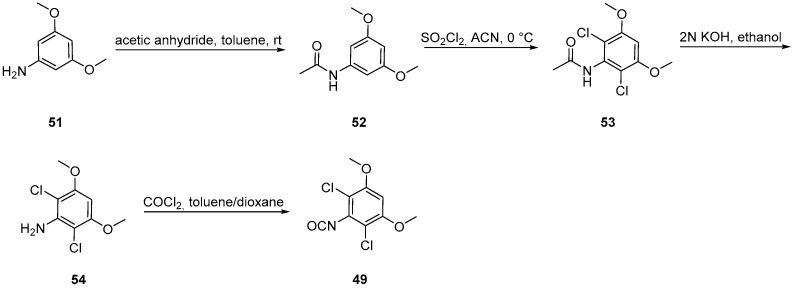
Preparation of the isocyanate **49** used for the synthesis of infigratinib.

The synthon **49** was prepared from the corresponding amine reacting with phosgene. The preparation of amine starts from 3,5-dimethoxyaniline **51**, which is acetylated, chlorinated by excess of sulfuryl chloride and deacetylated under basic conditions (Scheme 7). The authors stated that the monochlorination of compound **52** could be obtained by using a substoichiometric amount of sodium chlorate [42]. 

#### 3.1.6. Umbralisib

Umbralisib (**69**, brand name Ukonik^®^) is a phosphatidylinositol-3-kinase (PI3K) inhibitor approved for patients with relapsed or refractory indolent lymphoma [43]. It was launched by TG Therapeutics [44]. 

Umbralisib differs in chemical structure from compounds of the same class and shows a better pharmacokinetic profile [45]. This can be explained also by the higher number of fluorine atoms on the aromatic rings. The convergent synthesis [46] of umbralisib is accomplished by coupling two synthons (**60** and **66**). 

**Scheme 8:**
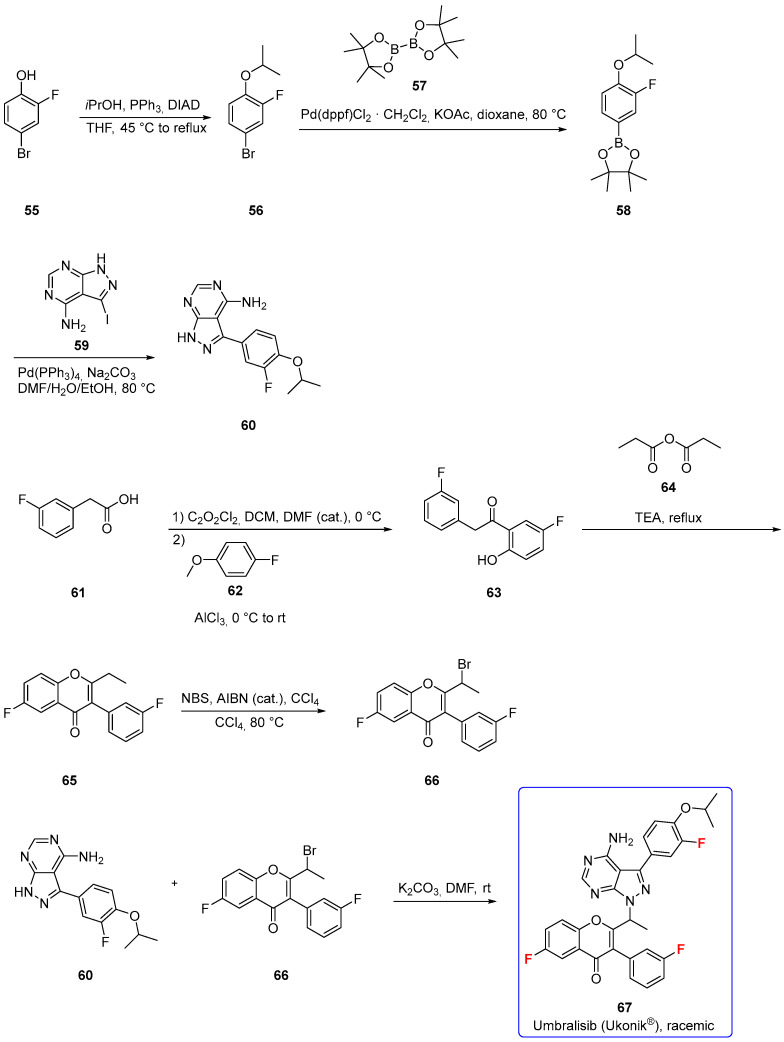
Synthetic scheme for the preparation of the racemic form of umbralisib (**67**) [46].

The synthetic pathway for the preparation of the two synthons and for racemic umbralisib (**67**) is shown in Scheme 8.

The synthesis of synthon **60 [46]** originates from the introduction of an isopropyl group on 4-bromo-3-fluorophenol via the classic Mitsunobu process. Then, the boronic ester **58** is formed via a Suzuki–Miyaura reaction with bis(pinacolato)diboron (**57**) catalyzed by Pd(dppf)Cl_2_. The subsequent Suzuki–Miyaura between **58** and 3-iodo-l*H*-pyrazolo [3, 4-*d*]pyrimidin-4-amine (**59**) catalyzed by Palladium-tetrakis(triphenylphosphine) afforded **60**.

For the second synthon (**66**), fluorophenylacetic acid was treated with oxalyl chloride, AlCl_3_ and 4-fluoroanisole (**62**) to afford **63** in a Friedel-Crafts acylation. The cyclization of **63** to the stable chromen-4-one **65** was mediated by propionic anhydride (**64**) in basic conditions (triethylamine, TEA), followed by the mono-bromination operated by NBS/AIBN (2-2′-azobisisobutirronitrile) in CCl_4_.

The final product (**67**) is given by a nucleophilic attack of **60** on **66** under alkaline conditions (K_2_CO_3_) in DMF.

**Scheme 9:**
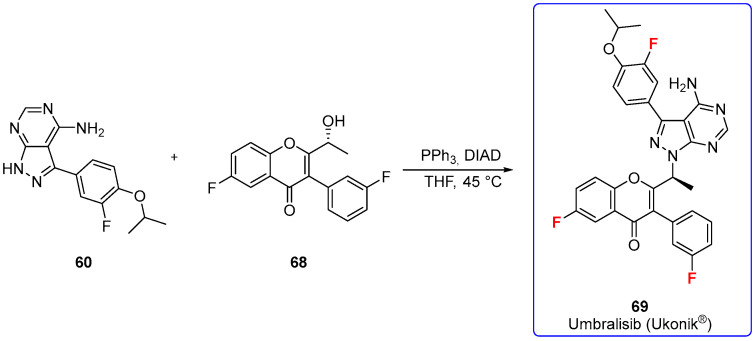
Synthetic scheme for the preparation of umbralisib (**69**) [46].

The stereoselective synthesis of umbralisib (Scheme 9) was achieved via the Mitsunobu process between the pyrrolic nitrogen of **60** and the secondary alcohol **68.** It proceeds with inversion of the configuration at the chiral center [46].

#### 3.1.7. Piflufolastat F-18

Piflufolastat F-18 (**77**, brand name Pylarify^®^) is an ^18^F-labeled diagnostic imaging agent that has been developed by Progenics Pharmaceuticals Inc. for positron emission tomography (PET) that targets prostate-specific membrane antigen (PSMA) [47].

Prostate cancer is the second-most common cancer diagnosed in men worldwide [47]; thus, Piflufolastat F-18 represents an important diagnostic tool.

**Scheme 10:**
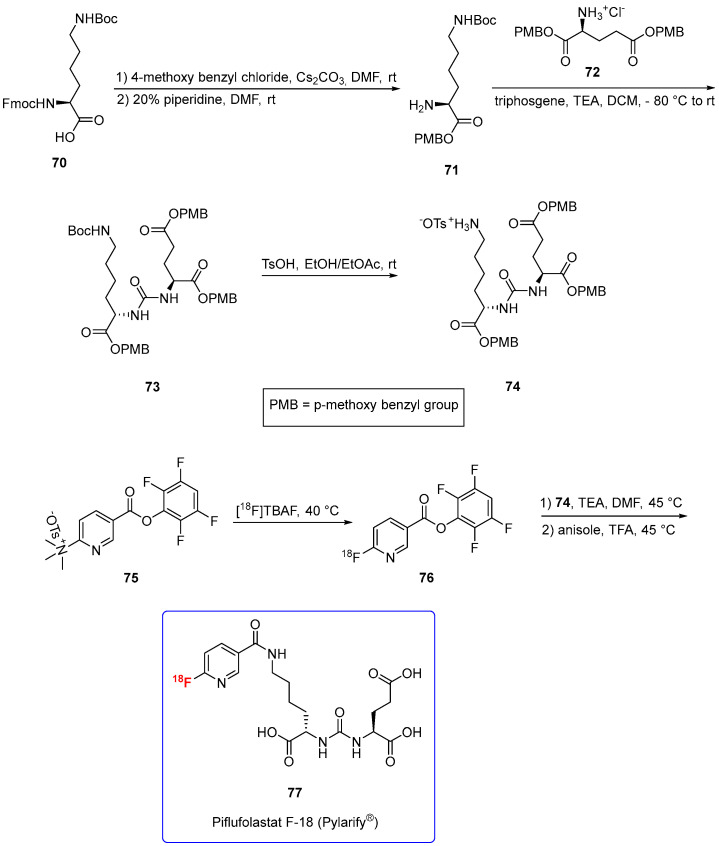
Synthetic scheme for the preparation of piflufolastat F-18 (**77**) [48].

The synthesis [48] (Scheme 10) originates from N_ε_-Boc-N_α_-Fmoc-l-lysine (**70**) that is firstly protected at acidic functionality with *p*-methoxy benzyl chloride, and secondly, Fmoc is cleaved by using a 20% piperidine solution to afford the derivative **71**. Urea **73** was obtained by treating bis-4-methoxybenzyl-l-glutamate·HCl (**72**) with triphosgene and triethylamine at −80 °C, followed by in situ trapping of the isocyanate intermediate by the addition of **71**. The selective removal of BOC by *p*-toluenesulfonic acid afforded **74**. *N*,*N*,*N*-Trialkylpyridin-2-aminium salt (**75**) was transformed into the corresponding radiofluorinated compound **76** upon using [18F]tetrabutylammonium fluoride ([^18^-F]TBAF), which was, in turn, reacted with **74** in the presence of triethylamine. Lastly, the removal of the PMB groups afforded Piflufolastat F-18 (**77**).

**Scheme 11:**
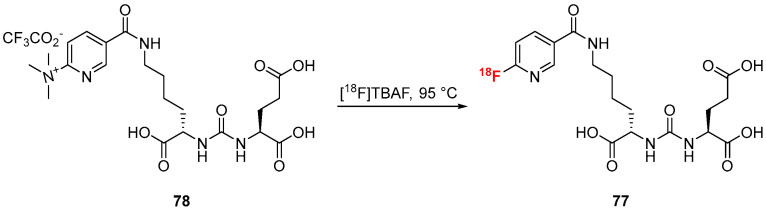
One-step pathway for the preparation of piflufolastat F-18 (**77**) [49].

Recently, a one-step pathway was proposed [49] (Scheme 11). The radiofluorination step occurred with no need to protect the carboxylic acid moieties.

The use of *N*,*N*,*N*-trialkylpyridin-2-aminium trifluoroacetate salt is advantageous in terms of yield. Moreover, the authors claimed that the process is notably optimized and allows for a full automation.

#### 3.1.8. Belzutifan

The Food and Drug Administration approved belzutifan (**92**, brand name Welireg^®^, Merck, Darmstadt, Germany), a hypoxia-inducible factor (HIF) inhibitor, for adult patients with von Hippel-Lindau disease who require therapy for associated renal cell carcinoma (RCC), central nervous system (CNS) hemangioblastomas or pancreatic neuroendocrine tumors (pNET) [50]. Structurally, it contains three fluorine atoms and three stereocenters.

The fluorine atom at the benzylic position of belzutifan may further decrease the electron density of the phenyl ring, resulting in a stronger noncovalent n→π*Ar electrostatic interaction with the Tyr281 lone pairs of the target protein, leading to an increased potency [51].

**Scheme 12:**
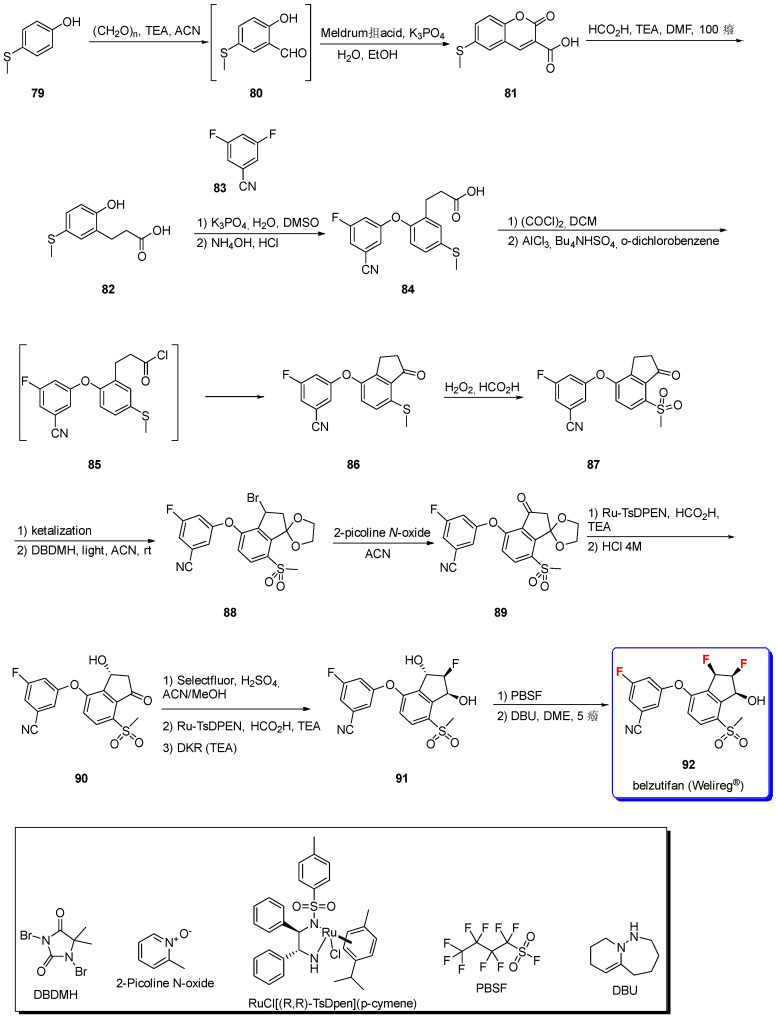
Synthetic pathway for belzutifan (**92**) [52].

The synthesis of belzutifan proceeds via six linear steps, starting from the indanone **87**, whose synthesis [52] is reported in Scheme 12.

The first step is the formylation of 4-(methylthio)-phenol (**79**) by the addition of paraformaldehyde to give aldehyde **80**. The condensation of **79** with Meldrum’s acid afforded the coumarin **81** in good yield (74%). Compound **81** was reduced and converted to propanoic acid derivative **82** via internal decarboxylation mediated by formic acid [53]. The subsequent S_N_Ar between **82** and 3,5-difluorobenzonitrile (**83**) in dimethyl sulfoxide (DMSO) formed the diaryl ether intermediate **84**, which was, in turn, activated to acyl chloride (**85**) by the addition of oxalyl chloride to yield the bicyclic system **86** via an internal Friedel-Crafts intramolecular reaction. Lastly, the oxidation of sulfide to sulfone afforded **87**.

The protection of ketone via ketalization and the selective bromination by (1,3-Dibromo-5,5-Dimethylhydantoin, DBDMH) via a photochemical process at room temperature afforded **88** [54]. The subsequent oxidation was carried out via using 2-picoline *N*-oxide as the oxidizing agent in Kornblum-type oxidation to give the ketone **89**. Reduction was carried out under Noyori conditions ((RuCl(*p*-cymene)[(*R*,*R*)-Ts-DPEN, RuTsDPEN, Scheme 12]), triethylamine and formic acid), and the following deprotection (accomplished by using hydrochloric acid) afforded the alcohol **90** [55,56].

Starting from **90**, the initial α-fluorination generated a mixture of fluorinated diastereomers, which were then subjected to Noyori asymmetric transfer hydrogenation conditions ((RuCl(*p*-cymene)[(*R*,*R*)-Ts-DPEN, RuTsDPEN], followed by a triethylamine (TEA)-promoted Dinamic Kinetic Resolution (DKR) triethylamine that afforded fluorodiol **91** as, essentially, a single diastereomer (dr > 99:1) [57].

Lastly, the authors identified perfluoro-1-butanesulfonyl fluoride (PBSF, Scheme 12) with 1,8-diazabicyclo[5.4.0]undec-7-ene (DBU, Scheme 12) as a uniquely effective reagent system for the deoxyfluorination step of fluorodiol to afford belzutifan (**92**). The reaction proceeded via the formation of the corresponding sulfonate on the alcoholic function and the subsequent S_N_2 attack from F^−^ ion with a corresponding inversion of the configuration [58].

### 3.2. Halogenated Antiviral Drugs Approved in 2021

2021 marked an important year for new anti-infective treatments and preventive therapies. Besides cabotegravir, which was approved for the treatment of HIV-1 [1], another halogenated drug (maribavir) was authorized for the treatment of cytomegalovirus infection.

#### 3.2.1. Cabotegavir

Cabotegravir (**105**, brand name Cabenuva^®^), developed by GlaxoSmithKline, is an integrase strand transfer inhibitor (INSTI) of the carbamoyl pyridone class. It is co-packaged with rilpivirine, available as long-acting injectable (LAI) formulations [59].

Halogen atoms play an important role in improving the metabolic stability and optimizing the pharmacological parameters, such as lipophilicity and permeability [60]. A similar role can be envisioned for the aromatic fluorine atoms of cabotegravir.

**Scheme 13:**
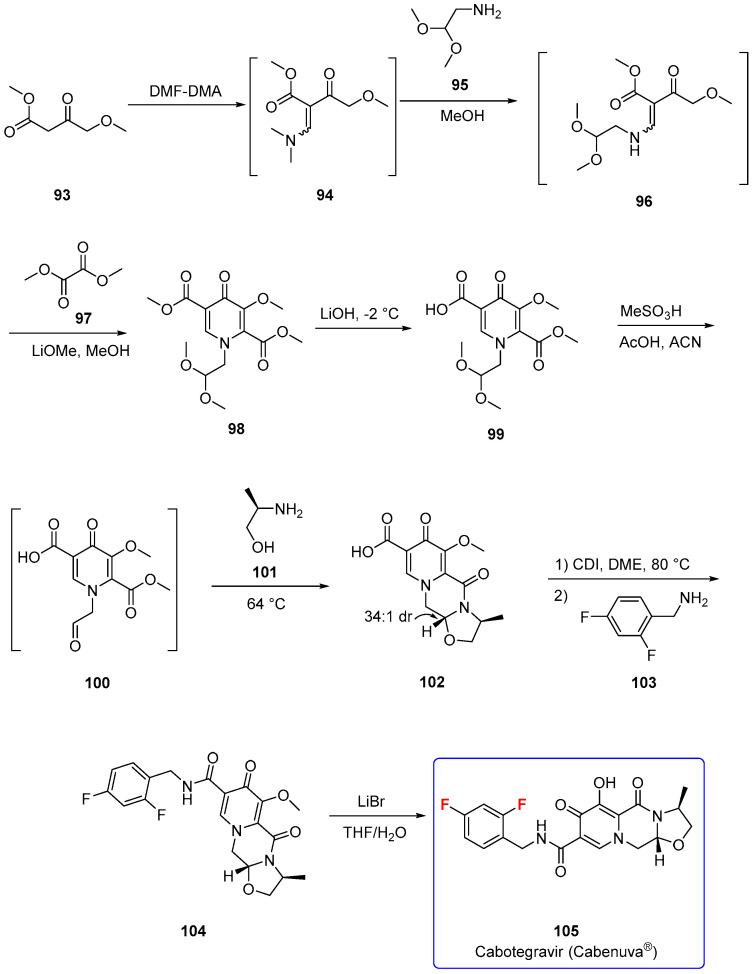
Synthetic pathway for cabotegravir (**105**) [61].

The synthesis of cabotegravir [61] (Scheme 13) originated from β-ketoester **93**, which, treated with *N*,*N*′-dimethylformamide dimethyl acetal (DMF-DMA), afforded enamine **94**. Compound **94** was mixed with aminoacetaldehyde dimethyl acetal (**95**) and MeOH to provide vinylogous system **96**, which was treated directly with dimethyl oxalate (**97**) to afford pyridone **98**. The selective hydrolysis at the desired ester moiety was carried out by using LiOH at low temperature to give the carboxylic acid **99**. The acetal deprotection via MeSO_3_H/AcOH afforded the aldehyde **100**, which was converted into the tricyclic ring **102** with a diastereomeric excess 34:1 upon the addition of (*S*)-alaninol (**101**) at 64 °C. The activation of the carboxylic acid via 1,1′-Carbonyldiimidazole (CDI) followed by the addition of 2,4-difluorobenzylamine (**103**) yielded the amide **104** that was converted into cabotegravir (**105**) by using LiBr as a deprotecting agent. Alternative ways for the demethylation steps have been reported. In particular, the use of magnesium salts (MgCl_2_, MgBr_2_ and MgI_2_) provide complete demethylation [62].

#### 3.2.2. Maribavir

Maribavir (**110,** brand name Livtencity^®^), available as an oral therapy, is owned by Takeda Pharmaceuticals and is indicated for the treatment of adults and pediatric patients with post-transplant cytomegalovirus (CMV) infection/diseases [63]. The drug inhibits viral DNA synthesis through the blocking of terminal DNA processing [64].

**Scheme 14:**
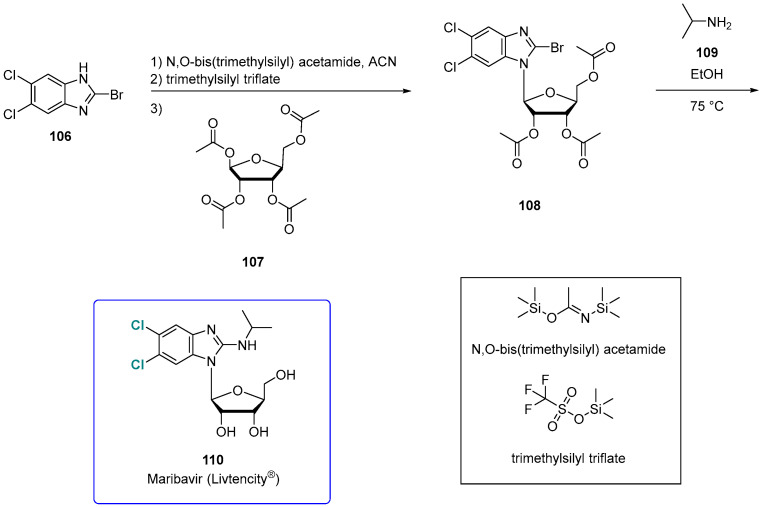
Synthetic pathway for maribavir (**110**) [65].

Chemically maribavir is a benzimidazole riboside, and its synthetic preparation is shown in Scheme 14. The synthesis [65] proceeds via trimethylsilyl-mediated *N*-glycosidation. It starts from 2-Bromo-5,6-dichlorobenzimidazole (**106**), which was combined with N,O-bis(trimethylsilyl) acetamide in acetonitrile. After the addition of trimethylsilyl triflate, the protected sugar (1,2,3,5-tetra-*O*-acetyl-l-ribofuranose) was added (**107**) to form the bromo derivative **108** that, upon treatment with isopropylamine (**109**), afforded maribavir (**110**) in a good yield (60%).

### 3.3. Halogenated Drugs Approved in 2021 for the Treatment of Multiple Sclerosis

One drug (ponesimod, Ponvory^®^), a selective sphingosine-1-phosphate receptor 1 (S1P1) [66], was approved for relapsing multiple sclerosis (RMS) [1]. MS is a complex, most likely autoimmune-mediated inflammatory neurodegenerative disease of the central nervous system (CNS). In Germany, an estimated 200,000 people suffered from such disease in 2014 [67], and this number increased to 250,000 in 2021 [68].

#### Ponesimod

Ponesimod (118, brand name Ponvory^®^) is a glycerol-based derivative, and it contains an atom of chlorine.

A SAR study showed that a 3-substituent next to the phenoxy group of the benzylidene moiety improved the activity towards Sphingosine-1-phosphate (S1P). A chloro or a methyl were the preferred substitutions [69].

**Scheme 15:**
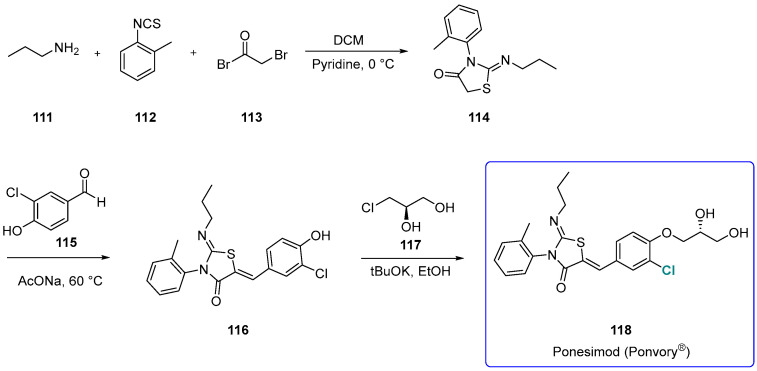
Synthetic pathway for ponesimod (**118**) [70,71].

The synthesis reported in Scheme 15 [70,71] starts with the preparation of thiazolidin-4-one **114** via mixing propylamine (**111**), o-tolyl isothiocyanate (**112**) and 2-bromoacetyl bromide **113** in the presence of pyridine, which has shown superior regioselectivity in the ring closure process when compared to triethylamine [71]. The regioselectivity of the cyclization is not only influenced by the reaction conditions and the choice of the halo acetic acid derivative but also by the nature of the two substituents of the so-formed thiourea intermediate [69]. The subsequent nucleophilic attack from the acidic proton on the thiazolidin-4-one ring to 3-chloro-4-hydroxybenzaldehyde (**115**) in the presence of sodium acetate led to the formation of phenol **116**. Lastly, the substitution on the phenolic position with (*R*)-3-chloropropane-1,2-diol (**117**) in alkaline environment afforded ponesimod (**118**).

### 3.4. Halogenated Drugs Approved in 2021 for the Treatment of Migraines

Migraine and severe headaches are a serious public health issue in the United States, women of childbearing age and those of lower socioeconomic status being the most affected groups. Socioeconomic disadvantages are also highly prevalent among those with headaches [72]. One halogenated drug (atogepant, Qulipta^®^) was approved in 2021 by the FDA for the treatment of episodic migraines [1].

#### Atogepant

Developed by AbbVie, atogepant (**130**, brand name Qulipta^®^) is an oral antagonist of calcitonin gene-related peptide (CGRP) receptors [73]. When CGRP binds to CGRP receptors, it causes pain, inflammation and vasodilation [74].

The trifluoro-benzene moiety in Atogepant has led to the higher affinity of atogepant (K_i_ = 0.015 nM) compared to the derivative bearing the unsubstituted benzene ring (K_i_ = 0.067 nM) [75].

**Scheme 16:**
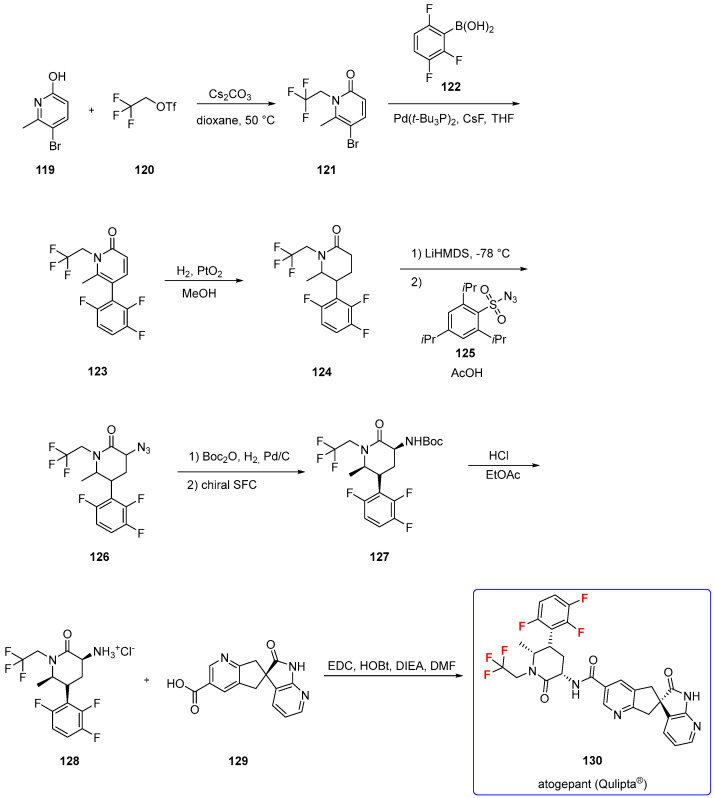
Synthetic pathway for atogepant (**130**) [76,77].

Chemically, it contains six fluorine atoms, and its synthesis [76,77] is shown in Scheme 16. The synthesis starts from 5-bromo-6-methylpyridin-2-ol (**119**), which is *N*-alkylated with 2,2,2-trifluoroethyl triflate to afford derivative **121**. The following Suzuki–Miyaura coupling with 2,3,6-trifluorophenylboronic acid (**122**) in the presence of Pd(t-Bu_3_P)_2_ and CsF afforded the diaryl derivative **123**. Catalytic hydrogenation (PtO_2_) in MeOH of **123** gave the piperidinone **124**, which was firstly lithiated by LiHMDS and, secondly, converted to the corresponding azide by using 2,4,6-triisopropylbenzenesulfonyl azide (**125**) to give **126**. The concomitant reduction to amine and BOC protection of intermediate **126** followed by Supercritical fluid chromatography (SFC) yielded the intermediate **127**, which underwent BOC removal (**128**) and condensation with carboxylic acid synthon **129** [76] to afford atogepant (**130**). Different routes for the preparation of synthon **129** have been proposed [77]. One selected pathway (the most straightforward considering the number of steps) is described in Scheme S3.

### 3.5. Halogenated Drugs Approved in 2021 for the Treatment of Vasculitis

Vasculitis, defined as a noninfectious inflammatory disorder of blood vessels, can affect different type of vessels in any organ [78]. Vasculitis could be associated with a relevant burden of mortality and morbidity if not recognized early and treated. Moreover, despite their rarity, their incidence and prevalence seem to be increasing in the last decade, partially because of an improved diagnosis and management of vasculitis from physicians [79].

One halogenated drug (avacopan, Tavneos^®^) was approved in 2021 for the treatment of severe active antineutrophil cytoplasmatic-associated vasculitis in combination with glucocorticoids [1].

#### Avacopan

Developed by ViforPharma, avacopan (**140**, brand name Tavneos^®^) is a selective inhibitor of the complement C5a receptor C5aR1. Avacopan arrests the ability of those cells to create damage in response to C5a activation, which is known to be the driver of antineutrophil cytoplasmic antibody (ANCA) vasculitis [80].

From a chemical point of view, avacopan contains four fluorine atoms (three on a trifluoromethyl group and one on the aromatic ring).

**Scheme 17:**
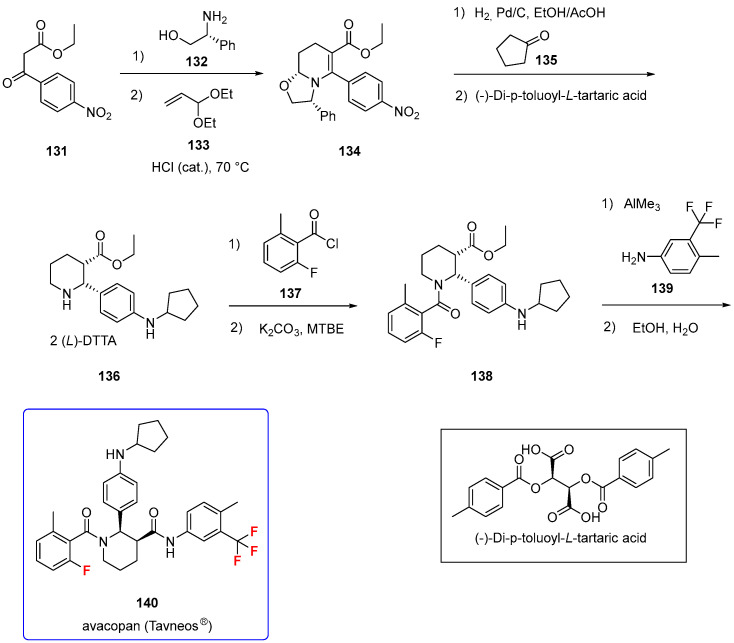
Synthetic pathway for avacopan (**140**) [81].

The synthetic pathway [81] is shown in Scheme 17, it starts from ketoester **131**, which forms the tetrahydro-oxazolo pyridine derivative **134** upon mixing with (*R*)-2-amino-2-phenylethan-1-ol (**132**) acrolein diethyl acetal (**133**) in the presence of catalytic HCl. Compound **134** is then subjected to hydrogenation (Pd/C) and treatment with cyclopentanone (**135**), followed by chiral resolution mediated by (-)-Di-p-toluoyl-l-tartaric acid, in order to form di-tartaric salt (DTTA) **136**. The nucleophilic substitution of piperidinic nitrogen of **136** with 2-fluoro-6-methylbenzoyl chloride (**137**) gave amide **138**, which was finally transformed into avacopan (**140**) by the addition of 4-methyl-3-(trifluoromethyl)aniline (**139**) in a AlMe_3_-mediated amidation.

### 3.6. Halogenated Drugs Approved in 2021 for the Treatment of Cardiovascular Disease

Cardiovascular diseases (CVDs) have been among the major causes of morbidity and mortality in developed countries over the last several decades [82]. One drug, vericiguat (Verquvo^®^), available in tablets was approved by the FDA to reduce the risk of cardiovascular death and heart failure hospitalization in certain high-risk patients [1].

#### Vericiguat

Launched by Bayer and Merck, vericiguat (150, brand name Verquvo^®^) directly stimulates soluble guanylate cyclase (sGC), independently of and synergistically with nitric oxide (NO), to produce more cyclic guanosine monophosphate (cGMP), leading to smooth muscle relaxation and vasodilation, which may improve cardiac function [83].

Vericiguat (having two fluorine atoms, one of which is in *meta* position to nitrogen of the pyridine ring) demonstrates superior pharmacokinetic properties compared to its monofluorinated precursor [84].

**Scheme 18:**
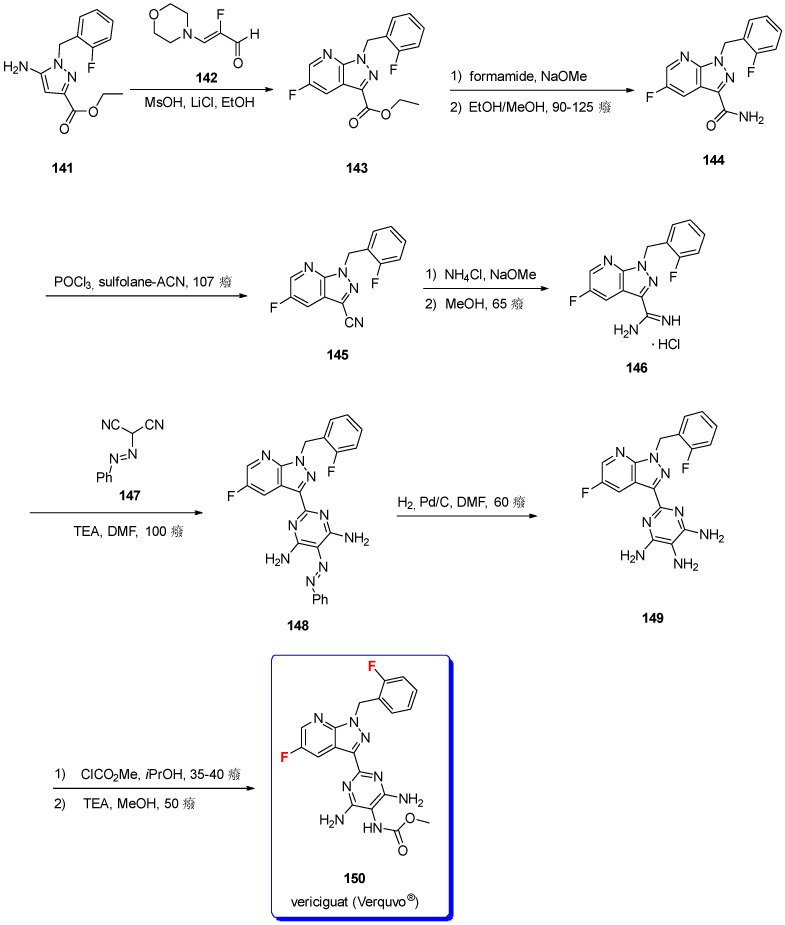
Synthetic pathway for vericiguat (**150**) [85].

The synthesis of vericiguat [85] is shown in Scheme 18. It starts with the condensation of 5-amino-1*H*-pyrazole (**141**) with aldehyde **142** to afford intermediate ester **143**. The subsequent formation of the primary amide (**144**) by using formamide and sodium methoxide was followed by the dehydration of amide to nitrile **145** via POCl_3_. Nitrile **145** was converted to amidine **146** upon the addition of ammonium chloride in a basic environment. Then, the addition of malononitrile **147** to **146** afforded the pyrimidine-4,5,6-triamine intermediate **148**. Catalytic hydrogenation was employed to remove the diazinyl protection, thus affording **149**. Lastly, the 5-NH_2_ group of **149** reacted with methyl chloroformate, followed by an alkaline workup, to afford vericiguat (**150**).

## 4. Conclusions and Future Perspectives

In this work, the role of halogens (in particular, fluorine and chlorine) in the recent drug discovery processes was discussed. Moreover, we listed all the halogen-containing drugs that were approved by FDA in 2021.

For each compound, the biological activity and the chemical synthesis were provided.

By analyzing our data, it appears that fluorine and chlorine substituents are becoming more and more popular given the role they play from a physicochemical and pharmacodynamic standpoint.

**Figure 3:**
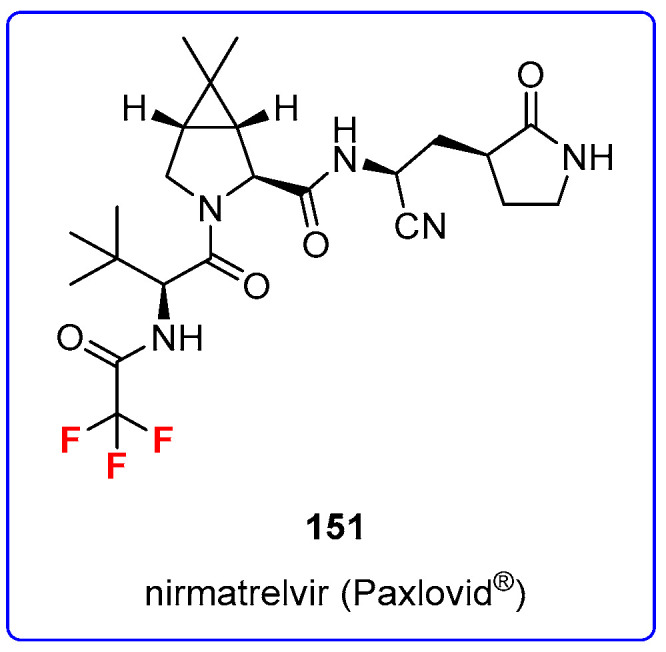
Chemical structure of nirmatrelvir (**151**).

Outside the scope of the present review but deserving a mention is the recently launched molecule nirmatrelvir (compound **151**, Figure 3) in the fight against the SARS-CoV-2 pandemic. It is a fluorine-containing prodrug developed by Pfizer. Compound **151** is an orally active 3C-likeprotease inhibitor in which “the trifluoroacetamide stood out in its ability to permeate the gut barrier in assays”, says a research team from Pfizer [86].

Nirmatrelvir (PF-07321332) plus ritonavir is a combination therapy that has the brand name of Paxlovid^®^ [87].

This example, together with others described throughout this work, reinforces our belief that plenty of halogenated structures will be serving in the future as treatments for several diseases.

## Figures and Tables

**Table 1:** Effect of fluorine on a 3-piperidinylindole series endowed with antipsychotic activities. *^a^* Affinities at a human-cloned 5-HT_2A_ receptor. *^b^* Acid dissociation constant for the piperidinic-NH. *^c^* Bioavailablity (%) in rats.

	5-HT_2A_ *^a^* (nM)	p*K*_a_ *^b^*	F (%) *^c^*
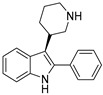	0.99	10.4	Poor
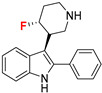	0.43	8.5	18
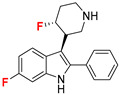	0.06	-	80

## Data Availability

Not applicable.

## Supplementary Materials

The following are available online, Scheme S1: Alternative synthesis for tivozanib, Scheme S2: Alternative synthesis for tivozanib, Scheme S3: Alternative synthesis for the synthon S18, Figure S1: Chemical structure of boroxine S9 employed in an optimized synthesis of Sotorasib, Table S1: Table describing the names of the 14 halogenated molecules approved by FDA in 2021.
