# Supplementary material for: New Halogen-Containing Drugs Approved by FDA in 2021: An Overview on Their Syntheses and Pharmaceutical Use
Source: Molecules. 2022 Mar 2;27(5):1643. doi: 10.3390/molecules27051643 (PMC8912053; doi:10.3390/molecules27051643)
Supplement: Supplementary file 1 [file molecules-27-01643-s001.zip › molecules-1609033-supplementary.pdf]

SUPPORTING INFORMATION

New Halogen-containing drugs approved by FDA in 2021: An Overview on their Syntheses and Pharmaceutical Use

Davide Benedetto Tiz *, Luana Bagnoli, Ornelio Rosati, Francesca Marini, Luca Sancineto and Claudio Santi *

Group of Catalysis, Synthesis and Organic Green Chemistry, Department of Pharmaceutical Sciences, University of Perugia, Via del Liceo 1, Perugia 06100, Italy; luana.bagnoli@unipg.it (L.B.);
ornelio.rosati@unipg.it (O.R.); francesca.marini@unipg.it (F.M.); luca.sancineto@unipg.it (L.S.)
* Correspondence: davide.benedettotiz@unipg.it (D.B.T.); claudio.santi@unipg.it (C.S.)

Content:

Alternative synthesis for tivozanib (**Scheme S1**).	**Page 2**
Chemical structure of boroxine S9 employed in an optimized synthesis of Sotorasib (**Figure S1**)	**Page 2**
Alternative synthesis for the multi-kilogram production of melphalan flufenamide hydrochloric salt (**Scheme S2**)	**Page 3**
Alternative synthesis for the synthon SI18 (**Scheme S3**)	**Page 3**
Table describing the names of the 14 halogenated molecules approved by FDA in 2021 (**Table S1**)	**Pages 4-5**

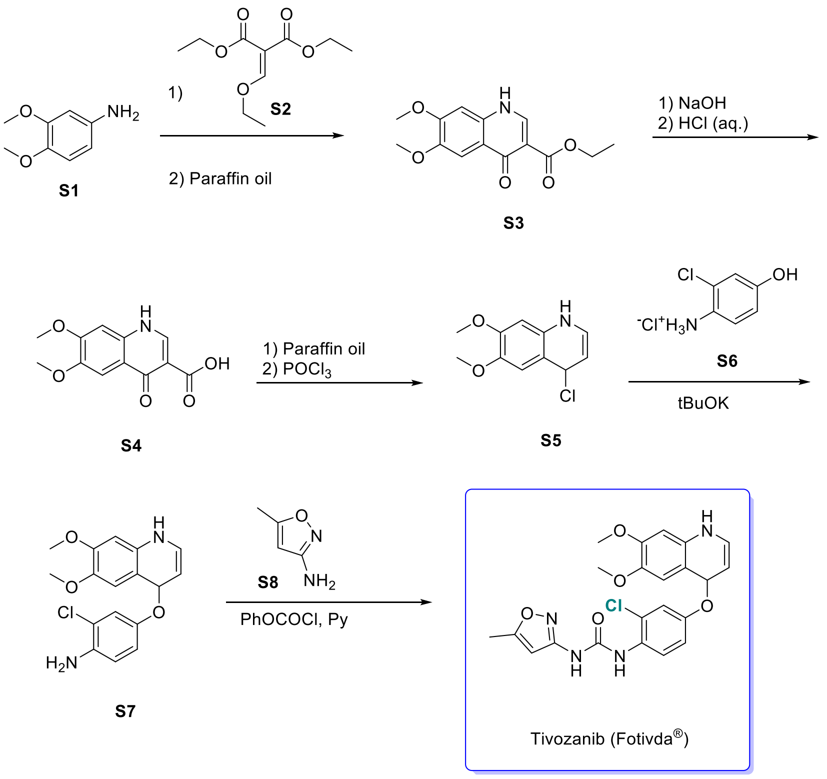

Scheme S1. Alternative synthesis for tivozanib [28].

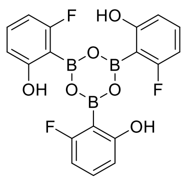

S9

Figure S1. Chemical structure of boroxine **S9** employed in an optimized synthesis of Sotorasib [31].

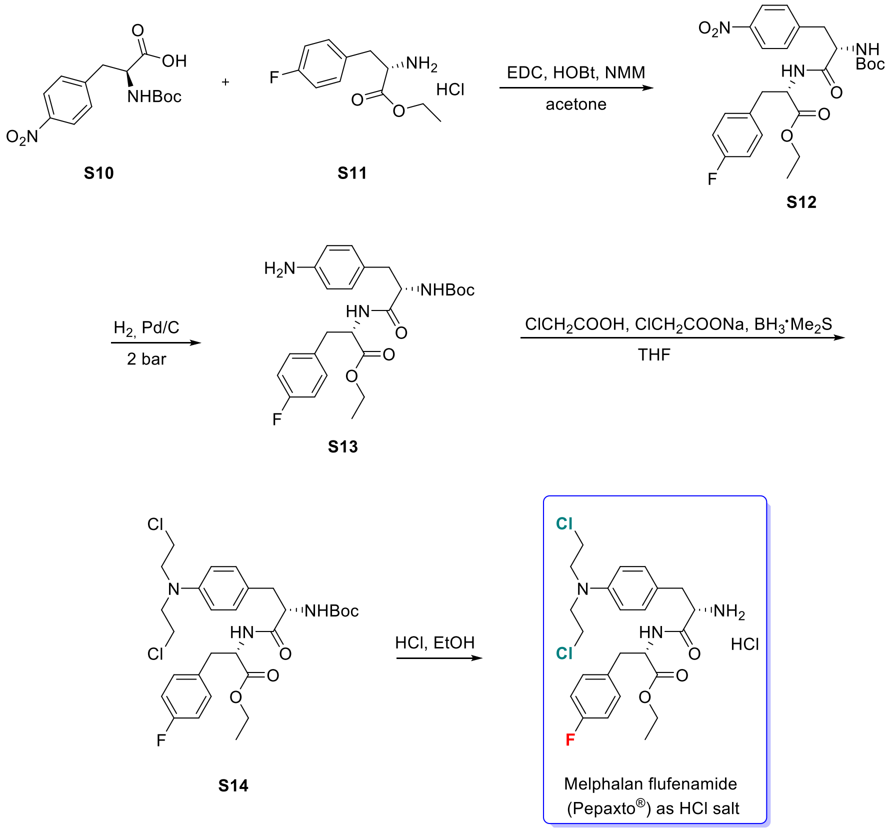

Scheme S2. Alternative synthesis for the multi-kilogram production of melphalan flufenamide hydrochloric salt [34].

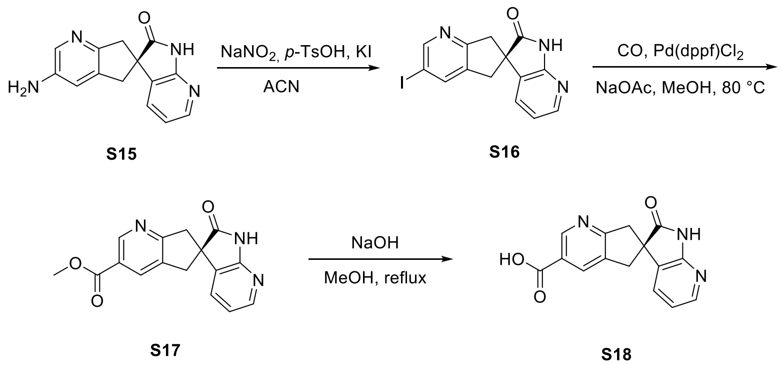

Scheme S3. Alternative synthesis for the synthon **S18** [77].

Name	Chemical structure	IUPAC name
**Tivozanib**	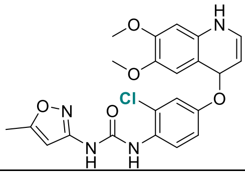	1-(2-chloro-4-((6,7-dimethoxy-1,4-dihydroquinolin-4-yl)oxy)phenyl)-3-(5-methylisoxazol-3-yl)urea
**Sotorasib**	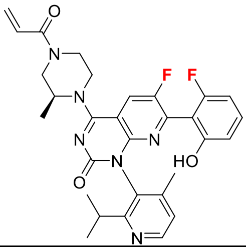	4-((S)-4-acryloyl-2-methylpiperazin-1-yl)-6-fluoro-7-(2-fluoro-6-hydroxyphenyl)-1-(2-isopropyl-4-methylpyridin-3-yl)pyrido[2,3-d]pyrimidin-2(1H)-one
**Melphalan flufenamide**	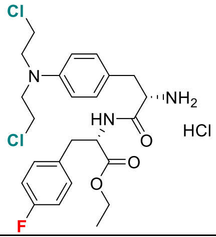	ethyl (S)-2-((S)-2-amino-3-(4-(bis(2-chloroethyl)amino)phenyl)propanamido)-3-(4-fluorophenyl)propanoate (HCl salt)
**Asciminib**	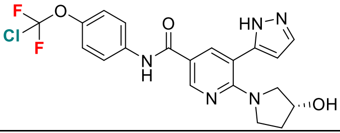	(R)-N-(4-(chlorodifluoromethoxy)phenyl)-6-(3-hydroxypyrrolidin-1-yl)-5-(1H-pyrazol-5-yl)nicotinamide
**Infigratinib**	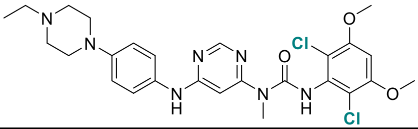	3-(2,6-dichloro-3,5-dimethoxyphenyl)-1-(6-((4-(4-ethylpiperazin-1-yl)phenyl)amino)pyrimidin-4-yl)-1-methylurea
**Umbralisib**	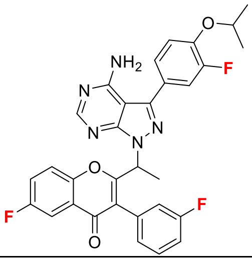	2-(1-(4-amino-3-(3-fluoro-4-isopropoxyphenyl)-1H-pyrazolo[3,4-d]pyrimidin-1-yl)ethyl)-6-fluoro-3-(3-fluorophenyl)-4H-chromen-4-one
**Piflufolastat F-18**	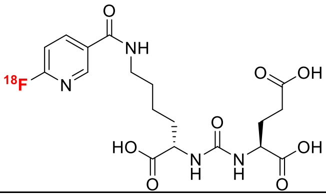	(((S)-1-carboxy-5-(6-(fluoro-18F)nicotinamido)pentyl)carbamoyl)-L-glutamic acid
**Belzutifan**	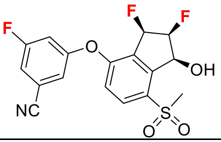	3-(((1S,2S,3R)-2,3-difluoro-1-hydroxy-7-(methylsulfonyl)-2,3-dihydro-1H-inden-4-yl)oxy)-5-fluorobenzonitrile
**Cabotegavir**	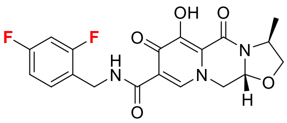	(3S,11aR)-N-(2,4-difluorobenzyl)-6-hydroxy-3-methyl-5,7-dioxo-2,3,5,7,11,11a-hexahydrooxazolo[3,2-a]pyrido[1,2-d]pyrazine-8-carboxamide

**Maribavir**	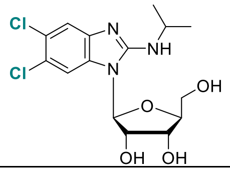	(2S,3S,4R,5S)-2-(5,6-dichloro-2-(isopropylamino)-1H-benzo[d]imidazol-1-yl)-5-(hydroxymethyl)tetrahydrofuran-3,4-diol
**Ponesimod**	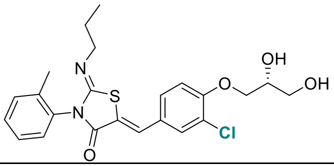	(Z)-5-((Z)-3-chloro-4-((R)-2,3-dihydroxypropoxy)benzylidene)-2-(propylimino)-3-(o-tolyl)thiazolidin-4-one
**Atogepant**	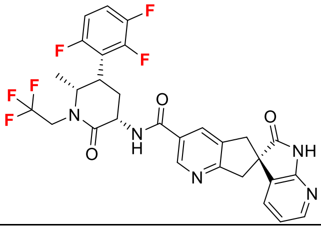	(S)-N-((3S,5S,6R)-6-methyl-2-oxo-1-(2,2,2-trifluoroethyl)-5-(2,3,6-trifluorophenyl)piperidin-3-yl)-2'-oxo-1',2',5,7-tetrahydrospiro[cyclopenta[b]pyridine-6,3'-pyrrolo[2,3-b]pyridine]-3-carboxamide
**Avacopan**	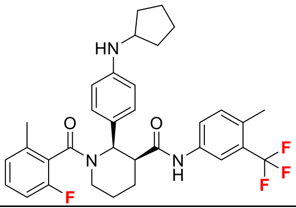	(2R,3S)-2-(4-(cyclopentylamino)phenyl)-1-(2-fluoro-6-methylbenzoyl)-N-(4-methyl-3-(trifluoromethyl)phenyl)piperidine-3-carboxamide
**Vericiguat**	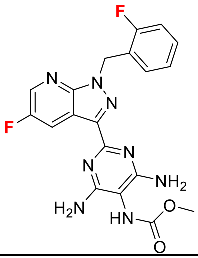	methyl (4,6-diamino-2-(5-fluoro-1-(2-fluorobenzyl)-1H-pyrazolo[3,4-b]pyridin-3-yl)pyrimidin-5-yl)carbamate

Table S1. Table describing the names of the 14 halogenated molecules approved by FDA in 2021. Their structures and IUPAC names are provided as well.
